# Preference and Values of Stroke Interventions, Kingdom of Saudi Arabia

**DOI:** 10.1155/2019/8502758

**Published:** 2019-04-01

**Authors:** Reem Alamri, Adel Alhazzani, Saeed A. Alqahtani, Hayfa Al-Alfard, Shahad Mukhtar, Khadejah Alshahrany, Faisal Asiri

**Affiliations:** ^1^College of Medicine, King Khalid University, Abha, Saudi Arabia; ^2^Neurology Section, Internal Medicine Department, College of Medicine, King Saud University, Riyadh, Saudi Arabia

## Abstract

*Background. *Acute ischemic stroke (AIS) occurs when there is a sudden occlusion of the arterial blood supply to part of the brain resulting in sudden focal neurological deficits. Recent major clinical trials of reperfusion therapy had proved the efficacy of timely stroke intervention to restore blood flow. Development of acute stroke protocols waiving the informed consent to obtain necessarily brain images or provide thrombolytic therapy is important to streamline and organize efforts to achieve the goal of early intervention and better functional outcome.* Objective. *This study aims to identify the preference and values of acute stroke interventions standard of care therapy without informed consent in the absence of surrogate decision-makers.* Methods.* A cross-sectional survey was conducted in the Kingdom of Saudi Arabia using an electronic questionnaire. The questionnaire addressed the patients' preference of acute stroke protocol waiving the informed consent for hyperacute brain images and delivering thrombolytic therapy or mechanical thrombectomy in absence of surrogate. All Saudi population aging from 18 to 65 years were invited to participate.* Results.* The study included 2004 participants with ages ranging from 18 to 65 years with mean age of 30.1 years. About 66% of the participants were females and 95% were Saudi. Overall, 90.5% of the participants agreed on performing computed tomography angiography (CTA) by the medical staff for the acute strokes without consenting followed by 79% for thrombolytic therapy, 70.8% for mechanical thrombectomy, and only 49.3% for acute lifesaving surgical intervention.* Conclusion.* Researchers found that the high percentage of participants had favorable response and positive perception toward providing acute stroke intervention and mechanical thrombectomy without informed consent. However, the study showed skeptical acceptance among participants regarding invasive surgical measures.

## 1. Background

Acute ischemic stroke (AIS) occurs when there is a sudden occlusion of the arterial blood supply to part of the brain manifested by focal neurological deficits [1]. It is life-threatening condition that needs an immediate intervention to minimize the neurological complications of the stroke [2]. More than 750,000 stroke cases occur every year in the United States, making it the fifth leading cause of death and the leading cause of disability [3]. Strokes have tremendous economic burden and devastating effect on the quality of life.

Stroke in Saudi Arabia is considered a major challenge to the health care system. In a recent published study analysis, the stroke incidence in Saudi Arabia is around 30 cases per 100,000 population [4] while a more recent study showed even higher incidence rate of first-time stroke of 57.64 per 100,000 person-years [5] with trends toward no declining in all mortalities and permanent disabilities [6] This study is a national survey to inform decision-makers on the current magnitude of the epidemic. The data was obtained from the nationwide physiotherapy departments that provide rehabilitation therapy to the stroke patients.

Development of clinical practice guidelines involves making trade-offs between desirable and undesirable consequences of alternative management strategies. Although the relative value of health states to patients should provide the basis for these trade-offs, few guidelines have systematically summarized the relevant evidence. We conducted a systematic review relating to values and preferences of patients considering antithrombotic therapy [7, 8]. Patient values and preferences regarding thromboprophylaxis treatment appear to be highly variable. Participant responses may depend on their prior experience with the treatments or health outcomes considered as well as on the methods used for preference elicitation. It should be standard for clinical practice guidelines to conduct systematic reviews of patient values and preferences in the specific content area [9–11].

In the treatment of stroke, the time factor is so important so that every one-minute delay in the intervention causes two million cells to die, because of which early stroke interventions including CT angiography, thrombolysis, and thromboectomy are the standard of care recently because of the better outcome to the patient.

Most of hospitals policies recommend taking consent before any intervention, but in many occasions, stroke patients have acute cognitive, or communication impairments which can partially or completely affect their ability to provide an informed consent which causes delay in the intervention and further worse outcome. Therefore, it is important to conduct this study to identify the preference and values of the public toward the emergency interventions of stroke and their preference on a physician intervention without an informed consent in the absence of a surrogate.

## 2. Methodology

A cross-sectional survey was conducted using an electronic questionnaire all over the kingdom of Saudi Arabia. The questionnaire was developed by the research team after intensive literature review and experts consultation for items and applicability of the tool. Any suggestions for modifications or changes were firstly discussed in groups and then final decision was reached till having this final format which was then transferred into electronic online format and all Saudi population aging from 18 to 65 years were invited to participate. The tool covered general personal characteristics including age, gender, education, residence, and income. Family history of stroke and intervention were also covered in the tool. The third section focused on the preference and values of stroke patients and their families related to the emergency intervention of stroke and their preference on a physician intervention without an informed consent in the absence of a surrogate decision-maker for all available types of intervention procedures including radiological, mechanical thrombectomy, medical, and surgical procedures using 5-point Likert scale ranging from strongly disagree till strongly agree. A total sample of 2004 responders was obtained and then data were extracted for analysis.

## 3. Data Analysis

After data were extracted, they were revised, coded, and fed to statistical software IBM SPSS version 20. All statistical analysis was done using two-tailed tests and alpha error of 0.05. P value less than or equal to 0.05 was considered to be statistically significant. Frequency and percent distribution were done for all data variables. The composite score for each intervention procedure preference item was calculated to have a composite mean for each which ranged from 1 to 5 and it was considered to have positive preference to do intervention directly without consensus if the composite mean was above 3.5. Multiple binary logistic regression analysis was used to detect the adjusted importance of the different predictors for having positive perception (preference).

## 4. Results

The study included 2004 participants whose ages ranged from 18 to 65 years with mean age of 30.1 years. About 66% of the participants were females and 95% were Saudi. Urban residence was recorded among 88.8% of the participants while 76.9% were university graduates. About 54% of the participants were unemployed while 40% work at either private or governmental sector. About 50% of the participants had low income (less than 5000 SR) (Table 1).

With regard to the stroke family history (Table 2), 49.5% of the respondents had one family member with history of acute stroke attack. Among those relatives, 65.8% had disability or died due to this attack while 60.8% received surgical treatment. About 84% of the participants agreed on physician intervention without consent of the relatives.

Table 3 shows the detailed preference and values of stroke intervention as recorded by the survey participants for each expected procedure. As for CT angiogram, 83.8% of the sampled population recorded their acceptance for emergency brain imaging.

In regard of considering thrombolytic therapy intervention, 82.0% of the sampled population recorded their acceptance for thrombolytic therapy. Approximately 78% of the sampled population recorded their acceptance for mechanical thrombectomy. Only 54.2% of study sample agreed for performing lifesaving surgical intervention.

**Figure 1:**
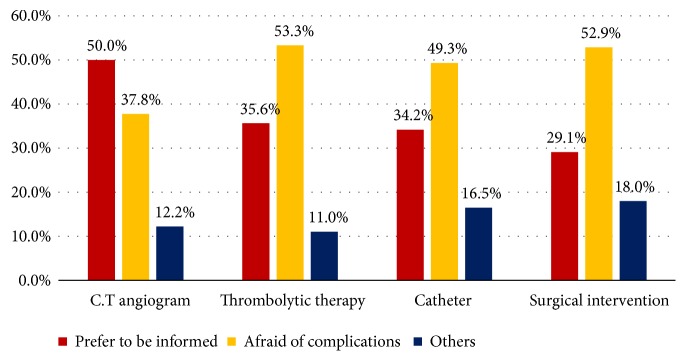
Causes of refusing undergoing the different stroke procedures directly without informing patients/relatives as recorded by general population shared in preference and values of stroke intervention, Kingdom of Saudi Arabia, 2018.

For those who recorded their disagreement for performing emergency stroke intervention without permission (Figure 1), the main causes were fear of complication in case of thrombolytic therapy, surgical intervention, and mechanical thrombectomy (53.3%, 52.9%, and 49.3%, respectively). Insisting of being informed was the major cause in case of need for CT angiogram (50%). More than half of the study sample (65.8%) have agreed to provide single consent form to authorize medical staff to perform all necessarily acute stroke intervention upon emergency room arrival.

In logistic regression model, older age was associated with increased probability for positive preference of acute stroke intervention without consent [43% (OR=1.43; 95% CI: 1.25-1.64)]. Females recorded lower positive preference rate by about 28% than males (OR =0.72; 95% CI: 0.56-0.93). Participants with family history of stroke recorded 18% more probability for accepting acute stroke intervention without consent (OR=1.18; 95% CI: 1.11-1.47).

## 5. Discussion

After reporting results researchers found that the participants had high scores and levels for positive perception regarding making the intervention for their stroke patients without informed consent specially if this intervention is effective, safe, and noninvasive in nature (thrombolytic therapy, radiology, and catheter). The response was less favorable among females and those who were highly educated who preferred to be informed firstly. The researchers recommended that if informed consent cannot be obtained and when legally authorized representative is not readily available it is justified to proceed with the appropriate acute diagnostic or therapeutic procedure in eligible patients according to the protocols and standards of care.

## 6. Conclusion

Researchers found that the high percentage of participants had favorable response and positive perception toward providing acute stroke intervention and mechanical thrombectomy without informed consent as those interventions are effective and time dependent. It is reasonable to proceed with the appropriate acute diagnostic or therapeutic procedure in eligible stroke patient when informed consent cannot be obtained from patients or surrogate. However, the study showed skeptical acceptance among participants regarding aggressive surgical measures like hemicraniectomy; therefore unless hemicraniectomy is performed emergently there should be thorough discussion about risk and benefits and an informed consent is required.

## Figures and Tables

**Table 1:** Personal data of general population shared in preference and values of stroke intervention, Kingdom of Saudi Arabia, 2018.

Personal data		No	%
Age in years	<20 years	194	9.7%
	20-	889	44.4%
	30-	460	23.0%
	40-	288	14.4%
	50+	173	8.6%

Gender	Male	671	33.5%
	Female	1333	66.5%

Nationality	Saudi	1916	95.6%
	Non-Saudi	88	4.4%

Residence	Urban	1779	88.8%
	Rural	225	11.2%

Educational level	Illiterate	7	.3%
	Primary	32	1.6%
	Secondary	423	21.1%
	University / more	1542	76.9%

Work	Not working / student	1085	54.1%
	Governmental sector	621	31.0%
	Private sector	188	9.4%
	Retired	110	5.5%

Monthly income	< 5000 SR	983	49.1%
	5000-10000 SR	381	19.0%
	> 10000 SR	640	31.9%

**Table 2:** Stroke family history among general population shared in preference and values of stroke intervention, Kingdom of Saudi Arabia, 2018.

Stroke family history		No	%
Have family member with acute stoke attacks	No	1013	50.5%
	Yes	991	49.5%

Caused disability or death (n=991)	No	339	34.2%
	Yes	652	65.8%

Received surgical interventions (n=991)	No	388	39.2%
	Yes	603	60.8%

Physician should directly interfere for therapy to avoid late intervention	Disagree	79	3.9%
	Neutral	237	11.8%
	Agree	1688	84.2%

If you or one of your relatives are the patient, physician should interfere directly without permission	Disagree	111	5.5%
	Neutral	199	9.9%
	Agree	1694	84.5%

**Table 3:** Discretion of preference and values of stroke intervention and telestroke survey among sampled population, Kingdom of Saudi Arabia, 2018.

Procedure	Item	Disagree		Neutral		Agree	
		No	%	No	%	No	%
CT angiogram	Degree of accepting this intervention	128	6.4%	196	9.8%	1680	83.8%
	Medical team should do needed imaging without permission in need	73	3.6%	135	6.7%	1796	89.6%
	Do you accept this procedure in case you or relative is the case?	69	3.4%	103	5.1%	1832	91.4%

Thrombolytic therapy	Degree of accepting this intervention	61	3.0%	299	14.9%	1644	82.0%
	Medical team should do needed imaging without permission in need	199	9.9%	300	15.0%	1505	75.1%
	Do you accept this procedure in case you or relative is the case?	136	6.8%	254	12.7%	1614	80.5%

Catheter	Degree of accepting this intervention	81	4.0%	364	18.2%	1559	77.8%
	Medical team should do needed imaging without permission in need	263	13.1%	395	19.7%	1346	67.2%
	Do you accept this procedure in case you or relative is the case?	189	9.4%	367	18.3%	1448	72.3%

Surgical intervention	Degree of accepting this intervention	336	16.8%	581	29.0%	1087	54.2%
	Medical team should do needed imaging without permission in need	535	26.7%	518	25.8%	951	47.5%
	Do you accept this procedure in case you or relative is the case?	454	22.7%	526	26.2%	1024	51.1%

Expert lack	Degree of accepting absence of experts in near hospital	301	15.0%	308	15.4%	1395	69.6%
	If patient is your relative, do you accept this?	307	15.3%	332	16.6%	1365	68.1%

## Data Availability

The data used to support the findings of this study are available from the corresponding author upon request.
